# Less improvement in knee function and higher rates of dissatisfaction in the short-term following total knee arthroplasty in people with mild radiographic arthritis

**DOI:** 10.1007/s00402-022-04564-5

**Published:** 2022-08-05

**Authors:** Yasser Khatib, Andrew Xia, Rui Liu, Justine M. Naylor, Ian A. Harris

**Affiliations:** 1 Orthopaedic Surgeon, Nepean Hospital, Sydney University, 2 Hope St, PO Box 949, Sydney, NSW 2750 Australia; 2 Nepean Hospital, Derby St, Penrith, NSW 2750 Australia; 3 Orthopaedic Department, South Western Sydney Clinical School, UNSW, Whitlam Orthopaedic Research Centre, Ingham Institute for Applied Medical Research, Liverpool Hospital, Locked Bag 7103, Liverpool BC, Sydney, NSW 1871 Australia

**Keywords:** Knee arthroplasty, Patient satisfaction, Treatment outcomes, Prognosis, Radiography, Oxford knee score

## Abstract

**Introduction:**

The purpose of this study was to assess if severity of radiographic changes of knee arthritis was associated with patient improvement after total knee arthroplasty (TKA). We hypothesised that patients with mild arthritis were more likely to report lower satisfaction, improvement in knee function and Oxford knee score (OKS) compared to patients with moderate or severe arthritis.

**Materials and methods:**

Secondary analysis of prospectively collected data from TKA patients of two arthroplasty centres with knee radiographs available for assessment of disease severity. Patients completed the Oxford knee score (OKS) and were asked to rate the global improvement in knee condition and their satisfaction at 6 months post-TKA. Bivariable analysis and multivariable regression models were used to test the association between disease severity and each outcome.

**Results:**

2226 patients underwent primary TKA and 3.6% had mild arthritis. Mean OKS improved from 17.0 (SD 18.0) to 38.0 (SD 8.1) 6 months after TKA. Two hundred and twenty-two patients (10%) reported ‘Poor’ or ‘Fair’ satisfaction, and 173 (8%) reported knee function was ‘Much worse’, ‘A little worse’ or ‘About the same’ 6 months post-TKA. Patients with mild arthritis showed improvement in OKS [mean improvement in OKS = 19 (SD 15)], but were significantly more likely to report dissatisfaction (OR = 3.10, 95% CI 1.62 to 5.91, *p* = 0.006), lack of improvement (OR = 4.49, 95% CI 2.38 to 8.47, *p* < 0.001) and lower OKS scores (− 3 points, 95% CI − 5.39 to − 0.85, *p* = 0.008) compared to patients with moderate to severe arthritis.

**Conclusions:**

While patients with mild radiographic arthritic changes improve after TKA, they were significantly more likely to report higher rates of dissatisfaction, less improvement in knee function and OKS compared to patients with moderate-severe grades of arthritis.

## Introduction

Total knee arthroplasty (TKA) provides a reliable long-term solution for the treatment of pain and improving function in patients with symptomatic knee arthritis. While the majority of patients improve, a proportion of patients do not. Many psychosocial, anthropometric, prosthetic and post-operative factors may affect outcome following TKA [1–7], but in many cases an obvious cause for failure to improve is not identified [8].

Pre-operative disease severity prior to TKA may be a factor in predicting post-operative outcome [9–16]. Disease severity is often measured radiographically and may influence the decision to recommend surgery. However, a relatively small proportion of TKA recipients are offered surgery for the treatment of symptoms that remain poorly controlled with less-invasive management despite having only mild radiographic changes of arthritis. This is because there is poor correlation between radiographic and clinical severity [16–19] and most patients show improvement after TKA.

The literature is divided as to the association between the preoperative severity of radiographic features of knee arthritis and the clinical outcomes after TKA. Some studies show better clinical outcomes after TKA (satisfaction, knee scores, pain levels or quality of life) in patients with moderate- to severe arthritis compared to patients with mild arthritis [10, 19–25], while others found no such association [26]. Many studies suffer from methodical flaws including small patient numbers [15], a focus on tibiofemoral signs of arthritis while neglecting the contribution of potentially severe patella-femoral joint (PFJ) arthritis, lack of multivariate statistical analysis and small cohort numbers [20].

The aim of this study was to assess the relationship between the pre-operative severity of radiographic features of arthritis in all compartments and clinical outcome 6 months after TKA. The hypothesis of this study is that TKA performed in patients with limited radiographic evidence of arthritis is more likely to be associated with less improvement in knee function.

## Materials and methods

Ethics approval was obtained from a lead Human Research Ethics Committee (HREC/18/Nepean/37). A dataset of total knee arthroplasty patients was compiled from the Australian Clinical Outcomes Registry National (ACORN) (www.acornregistry.org).

### Database

**Fig. 1:**
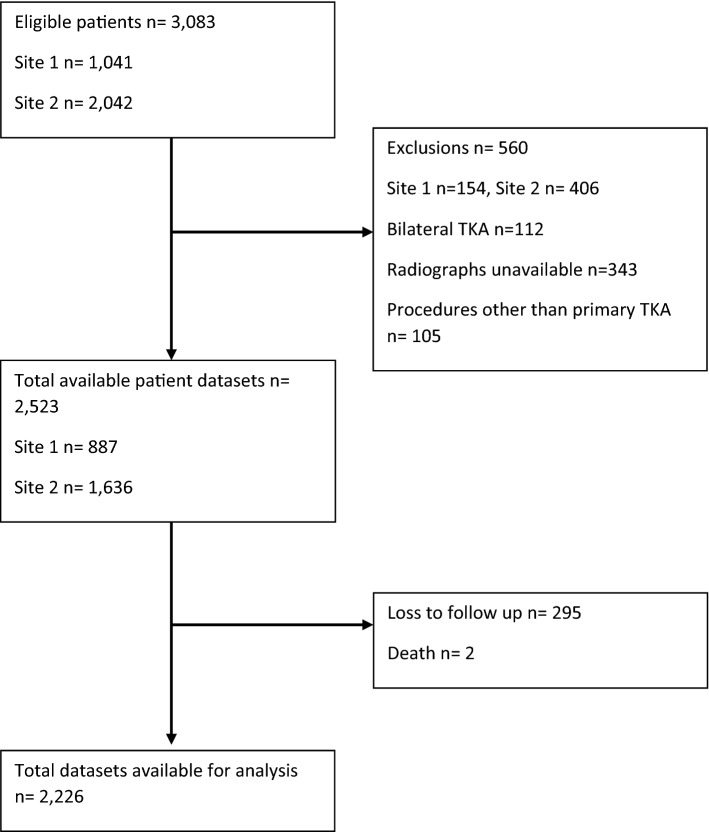
Flow chart of inclusion of patients in this study

ACORN is a multicentre hip and knee arthroplasty registry established in 2012 to collect and analyse patient demographics, surgical factors and 6-month clinical outcome measures in patients undergoing hip or knee arthroplasty. The ACORN registry does not conduct further follow-up beyond 6 months. Patients are enrolled into ACORN via an opt-out consent process. Data are collected directly from the patient as well as from the medical record. Data from the latter are extracted by a site coordinator after the patient is discharged. The study sample included adult patients who had elective, primary TKA surgery for any pathology in one of two participating high-volume arthroplasty centres, who had their data captured by the ACORN database from inception until April 2018. We excluded patients who did not have available radiographs and cases of bilateral TKA as the results of a well-functioning TKA on one side could mask the results of a poor TKA on the other side and vice versa. We also excluded patients who were lost to follow-up and, therefore, did not have 6-month post-operative outcomes collected. A flowchart of patient inclusion is detailed in Fig. 1.

The ACORN database included pre-operative data such as age, gender, BMI, baseline health status [American Society of Anesthesiologists (ASA) class] [27, 28] and baseline patient-reported measures such as OKS and EuroQol Health-Related Quality of Life: 5 Dimensions (EQ5D), perioperative and postoperative data including the occurrence of complications and clinical outcomes 6 months after surgery. Patient expectations of the level of pain (no pain, slight, moderate or severe pain) and knee function (no limitation, slight, moderate or severe limitation) after TKA were recorded preoperatively.

### Radiographic scoring

The two centres were selected as they routinely performed pre-operative knee radiographs including weight-bearing anteroposterior (AP), lateral, knee flexion (Rosenberg) radiographs and a skyline view on all patients. Two investigators (AX and RL) blinded to patient outcomes, independently assessed patient radiographs randomly divided between them. The tibiofemoral joint (TFJ) was assessed using the AP, Rosenberg and lateral views. The patellofemoral joint (PFJ) was assessed using the skyline and lateral views. The individual knee compartments affected by disease were recorded. From AP radiographs the Kellgren and Lawrence (KL) grading (0–4) was used to indicate the severity of disease affecting the TFJ. The severity of PFJ arthritis was recorded as nil, mild, moderate or severe. The overall severity of knee arthritis was assessed considering the radiographic evidence of arthritis in both TFJ and PFJ compartments. The final grade was given for the worst affected compartment as assessed on the Kellgren–Lawrence grade for the tibiofemoral joint and the joint space narrowing of the patellofemoral joint; and labelled as “Disease Severity” (DS) grade. A table of the radiographic assessment system used is shown in Table 1. The reliability and validity of the Disease Severity grades were not tested in this study.

**Table 1:** Grading systems used in the interpretation of patient radiographs and the development of a Disease Severity (DS) grade accounting for the worst affected knee compartment

Variable assessed	Interpretation
*Compartment affected*	
	No definite compartment involvement
	PFJ only
	Medial compartment only
	Lateral compartment only
	Medial and PFJ
	Lateral and PFJ
	Medial and Lateral
	Tricompartmental
*Kellgren and Lawrence grade*	
0	No radiographic features of OA are present
1	Doubtful joint space narrowing (JSN) and possible osteophytic lipping
2	Definite osteophytes and possible JSN on anteroposterior weight-bearing radiograph
3	Multiple moderate osteophytes, definite JSN, small cystic areas with sclerotic walls and possible deformity of bone contour
4	Large osteophytes, marked JSN, severe sclerosis and definite deformity of bone contour
*Patellofemoral joint space narrowing*	
0	Nil (no evidence of JSN)
1	Mild (evidence of possible JSN)
2	Moderate (definite evidence of JSN)
3	Severe (marked JSN)
Disease severity (DS) grade accounting for worst affected knee compartment (TFJ or PFJ)	
DS 1—no changes of disease	Highest grade: KL 1 or PFJ 0
DS 2—mild	Highest grade: KL 2 or PFJ 1
DS 3—moderate	Highest grade: KL 3 or PFJ 2
DS 4—severe	Highest grade: KL 4 or PFJ 3

After a period of training, the two assessors were compared for intra-observer and inter-observer reliability for the assignment of KL grade by recording their interpretation of 50 randomly selected patient x-ray sets assessed on two separate occasions one week apart. The unweighted *κ*-coefficient showed good reliability between the two observers (unweighted *κ*-coefficient, *κ* = 0.73 for KL grade).

### Outcomes

The hypothesis was tested using the following three outcomes: first, patient-rated satisfaction with TKA 6 months after surgery. Second, patient-rated improvement in knee function 6 months after surgery. Both outcomes were assessed using the UK patient-reported outcome measures (PROM) questions for satisfaction and global improvement [29]. For the satisfaction question, patients were asked “How would you describe the results of your operation?” with five options provided: ‘Excellent’; ‘Very good’; ‘Good’; ‘Fair’; or ‘Poor’. For improvement in knee function, the question asked was “Overall, how are the problems now with your knee on which you had surgery, compared to before your operation?” This question allowed one of the following five possible options: ‘Much better’; ‘A little better’; ‘About the same’; ‘A little worse’; and ‘Much worse’. Patients were classed as ‘Satisfied’ if they rated their results as ‘Good’; ‘Very good’; or ‘Excellent’. Patients were rated as ‘Improved’ if patients rated their knee problems as ‘A little better’ or ‘Much better’.

The third outcome was the Oxford knee score (OKS) which was measured pre-operatively and 6 months after surgery. The minimal important change (MIC) in OKS was considered to be an improvement of 9 points [30, 31].

### Statistical analysis

Descriptive statistics summarised the demographic and clinical patient characteristics. Statistical analysis was performed using disease severity grade (DS) as the radiographic variable of interest. Separate analysis was performed using the conventional KL grading system alone to assess the radiographic severity of arthritis, to compare the findings for consistency and to examine the difference when taking into account PFJ arthritis.

Unadjusted bivariable analyses (Pearson chi squared for categorical variables, and independent sample *t* test or analysis of variance for continuous variables) were used to examine associations between different variables and the outcome of interest. The significance threshold was set at *p* < 0.05. Missing data were considered missing-at-random and accounted for in statistical analysis by multiple imputation using chained equations to improve efficiency of the regression analyses. As the group of patients with mild disease was small in number, disease severity grade was dichotomized to simplify interpretation and allow sufficient statistical power, comparing patients with definite joint space narrowing in any compartment (DS 3 or 4) to patients with no or doubtful joint space narrowing (DS 1 or 2). Adjusted analysis using backward stepwise multivariable regression analyses using Akaike Information Criteria (AIC) on an imputed dataset was used for model selection, with the main predictor variable (disease severity) forced into the model, and separate model selections performed for each outcome: patient satisfaction, knee improvement or OKS. Covariates used were as follows: age, gender, BMI, ASA class; previous history of TKA on the opposite side, pre-operative anxiety or depression, occurrence of a complication; and patient pre-operative expectations of pain and function after surgery. The adjusted associations between disease severity and each outcome were calculated using pooled analyses from all imputed datasets.

Statistical analysis was performed using SPSS v26 (IBM SPSS Statistics for Windows, Version 26.0, 2019, Armonk, NY: IBM Corp) and R Statistical Environment for Computing.

The STROBE guidelines for observational studies were followed in reporting the results of this study.

## Results

From 3083 patients in the ACORN database who had TKA at one of the two participating hospitals during the study period, 2226 patients (72%) met the inclusion criteria and had a set of knee radiographs available for analysis and 6-month outcomes post-TKA. Patient demographics are summarised in Table 2; and patient outcomes before- and 6 months after surgery are summarised in Table 3. Most patients (*n* = 1650, 74%) expected ‘No’ or ‘Slight’ pain after TKA and ‘No’ or only ‘Slight’ limitation in function. Mean OKS and EQ VAS improved significantly after surgery, 10% (222 patients) reported dissatisfaction with TKA and 8% (173 patients) reported their knee condition did not improve.

**Table 2:** Patient demographics and health characteristics

	*n* or mean (SD)	% or (range)
Age	69 (8.99)	(36–92)
BMI	33 (7.25)	(10–65)
Gender		
Female	1461	66
Male	765	34
ASA class		
1 and 2	1043	47
3 and 4	712	32
Diagnosis		
Osteoarthritis	1999	90
Rheumatoid arthritis	15	0.7
Other inflammatory	4	0.2
Osteonecrosis	12	0.5
Traumatic arthritis	47	2
Previous TKA		
No	1622	73
Yes	570	26

*ASA* American society of anesthesiologists, *BMI* body mass index

**Table 3:** Patient-reported scores before and 6 months after surgery

	*n* or mean (SD or %)	Range
Pre-operative EQ VAS	63 (22.01)	0–100
Pre-operative OKS	17 (7.66)	0–47
EQ5D Anxiety/depression		
None-slight	1304 (59)	
Moderate-extreme	864 (39)	
EQ5D pain and discomfort		
None-slight	181 (8)	
Moderate-extreme	1984 (89)	
6-month EQ VAS	75 (17.96)	0–100
6-month OKS	38 (8.09)	3–48
Complications		
SSI	26 (1)	
MUA	7 (0.3)	
DVT	5 (0.2)	
PE	1 (0.04)	
Other	15 (0.7)	
Reopration on joint	14 (0.6)	
Pain expectation		
No pain	1063 (48)	
Slight pain	587 (26)	
Moderate pain	76 (3)	
Severe pain	4 (0.2)	
Function expectation		
No limitation	1069 (48)	
Slight limitation	581 (26)	
Moderate limitation	79 (4)	
Severe limitation	3 (0.1)	
Satisfaction with TKA		
Good–excellent	1987 (89)	
Poor-fair	222 (10)	
Improvement in knee function		
A little better- much better	2036 (92)	
Much worse- about the same	173 (8)	

*SSI* superficial site infection, *MUA* manipulation under anaesthetic, *DVT* deep vein thrombosis, *PE* pulmonary embolus

The pattern of joint involvement based on the radiographic features of arthritis is shown in Table 4. In assessment of the TFJ according to the KL grading system 122 patients (5.5%) received TKA with no or only mild radiographic changes of arthritis, but when taking into account the severity of the most affected joint compartment including the PFJ using the disease severity score (DS), 81 patients (3.6%) received TKA despite no or only mild radiographic features of arthritis on their preoperative radiographs. The DS grade was used in further analysis.

**Table 4:** Anatomical location and severity of affected knee compartment(s)

Anatomy or severity of arthritis	*n* = 2226 (%)
Anatomy of involved compartment	
No definite involvement	16 (0.7)
Lateral only	7 (0.3)
PFJ only	25 (1.1)
Medial only	43(1.9)
Medial and lateral	29 (1.3)
Lateral and PFJ	90 (4.0)
Medial and PFJ	388 (17.4)
Tricompartmental	1628 (73.1)
KL grade	
1	43 (1.9)
2	79 (3.5)
3	1096 (49.2)
4	1008 (45.3)
Isolated PFJ arthritis	25 (1.1)
Mild	7 (0.3)
Moderate	6 (0.3)
Severe	12 (0.5)
Disease severity grade accounting for worst affected compartment (TFJ and PFJ)	
1	27 (1.2)
2	54 (2.4)
3	886 (39.8)
4	1259 (56.6)

*KL* Kellgren and Lawrence grade, *PFJ* patellofemoral Joint, *TFJ* tibiofemoral Joint

### Unadjusted bivariable analysis

There were no significant differences between patients with osteoarthritis compared to all other pathologies for reported dissatisfaction [194 (9.8%) vs 8 (10.3%), *p* = 0.89], lack of improvement [149 (7.5%) vs 5 (6.4%), *p* = 0.72], OKS [38 (SD 8.12) vs 39 (SD 7.77), *p* = 0.63]. Therefore, further analysis was conducted considering all pathologies together.

There was an association between lower grades of disease severity with dissatisfaction and failure of improvement in knee function (Table 5). Similar relationship was present when assessing the association between lower KL grades and the occurrence of the outcome of dissatisfaction, failure to improve after TKA and lower mean post-operative OKS (Table 7).

**Table 5:** The unadjusted association between patient (a) dissatisfaction and (b) reported failure to improve knee function and (c) mean 6-month post-operative OKS with radiographic severity of knee arthritis

(a) Disease severity	Dissatisfied (%) (*n* = 222)	OR	95% CI for OR		*p* value
			Lower	Upper	
1	6 (22)	4.4	1.7	11.3	0.002
2	11 (20)	4.0	2.0	8.0	< 0.001
3	129 (15)	2.7	2.0	3.6	< 0.001
4	76 (6)	Reference			Reference

(b) Disease severity	Failure to improve (%) (*n* = 173)	OR	95% CI for OR		*p* value
			Lower	Upper	
1	6 (22)	5.7	2.2	14.5	< 0.001
2	14 (26)	6.9	3.6	13.4	< 0.001
3	93 (11)	2.3	1.7	3.3	< 0.001
4	60 (5)	Reference			Reference

(c) Disease severity	Mean OKS (SD)	Mean difference	95% CI of the mean difference		*p* value
			Lower	Upper	
1	35 (10.0)	3.9	− 0.1	7.9	0.06
2	36 (9.3)	2.9	0.2	5.5	0.03
3	37 (8.7)	1.8	1.1	2.5	< 0.001
4	39 (7.4)	Reference			Reference

### Adjusted analysis

The odds of unsatisfactory outcome or failure of improvement after TKA (Table 6) in those with mild radiographic changes of arthritis, adjusting for the covariates in the model were higher in the group of patients with mild disease severity compared to moderate to severe disease (DS 1 or 2 vs DS 3 or 4).

**Table 6:** Final regression models for the outcomes of (a) dissatisfaction (b) failure to improve after TKA and (c) the outcome of OKS at 6 months

(a) Variable	OR	95% CI for OR		*p* value
		Lower	Upper	
DS 1 or 2 (vs 3 or 4)	3.10	1.62	5.91	0.006
EQ VAS	0.99	0.98	1.00	0.005
Preop OKS	0.99	0.97	1.02	0.85
Complications				
Present	1.57	1.07	2.30	0.02
Expectation of function				
Moderate-severe limitation (vs no-slight limitation)	1.31	0.69	2.51	0.42

(b) Variable	OR	95% CI for OR		*p* value
		Lower	Upper	
DS 1 or 2 (vs 3 or 4)	4.49	2.38	8.47	< 0.001
Previous TKA				
Yes	0.62	0.41	0.93	0.02
Complications				
Present	1.73	1.14	2.64	0.01
Expectation of function				
Moderate-severe limitation (vs no-slight limitation)	1.21	0.53	2.72	0.66

(c) Variable	Coefficient	95% CI for coefficient		*p* value
		Lower	Upper	
DS 1 or 2 (vs 3 or 4)	− 3.12	− 5.39	− 0.85	0.008
Age				
Years	0.07	0.03	0.11	< 0.001
Gender				
Male	1.61	0.89	2.33	< 0.001
Complications				
Present	− 1.13	− 2.21	-0.06	0.01
Expectations of function				
Moderate-severe limitation (vs no or slight limitation)	− 0.89	− 2.83	1.04	0.38
EQ5D Usual activities				
Moderate-extreme difficulties (vs no or slight limitation)	− 0.41	− 1.30	0.47	0.36
EQ5D anxiety/depression				
Moderate-extreme difficulties	− 1.03	− 1.82	− 0.24	0.01
EQ VAS	0.03	0.02	0.05	< 0.001

Receiver Operator Characteristic (ROC) curves used to assess the discriminatory capacity of the regression model showed the area under curve (AUC) for the outcome of satisfaction was 0.61 and AUC was 0.64 for the outcome of improvement. Using the Hosmer and Lemeshow goodness of fit test the *p* values were 0.12 and 0.32, respectively, which indicate no evidence of poor fit of the model [32].

Regression analysis for the outcome of OKS 6 months after TKA showed significantly less improvement with mild disease severity (DS 1 or 2) with approximately three points less improvement compared to patients with more severe disease changes (DS 3 or 4) (Table 6).

## Discussion

While the overall rates of dissatisfaction (10%) and failure to improve (8%) were low, compared to patients with moderate or severe arthritic changes on their pre-operative radiographs, patients without those changes were significantly more likely to report dissatisfaction and failure to improve 6 months after surgery. The OKS also showed lower absolute scores and less improvement with mild arthritis; however, the magnitude of this difference was small and unlikely to be of clinical significance.

A possible explanation for these findings is that patients with mild arthritis have symptoms originating external to the affected knee. Previous studies have shown that pre-operative pain sensitization may be an important factor in the development of persistent pain after TKA [25]; others have shown that patient anxiety and depression were associated with increased rates of dissatisfaction and less improvement after TKA [19]. In this study we used the pain and the anxiety/depression components of the EQ 5D questionnaire pre-operatively to detect the group of patients who responded with ‘moderate’, ‘severe’ or ‘extreme’ to either of these questions. There were no significant associations between these groups of potentially at-risk patients with the development of dissatisfaction or failure to improve after TKA. Similarly, patient preoperative expectation of pain and function after TKA may be an important factor in achieving patient satisfaction [26]. In this study, patient expectations of levels of pain and function after TKA were examined; and no significant association was found between expectations and any of the outcomes used.

The findings of this study are consistent with other cohort studies that showed similar results of worse clinical outcomes and 2–3 times higher rates of dissatisfaction in patients with K-L grade 0–2 changes [10, 20, 21, 23–25].

This study reports on a relatively large overall sample size, whereas previous studies have only reported on relatively small number of patients ranging from 264 to 860 [25, 26]. The performance of TKA for limited radiographic changes of OA is an uncommon event (< 6%, *n* = 122 patients). Furthermore, the occurrence of dissatisfaction in this series is an uncommon event, reported in < 10% of patients (*n* = 222). Therefore, our analyses are based on a relatively small group of dissatisfied patients who has mild radiographic changes. A limitation of this analysis is that nearly 28% of patients who were recruited to the ACORN database who met the inclusion criteria either did not have available knee radiographs for analysis or were lost to follow-up during the period of 6 months after the surgery. While this is a limitation of the ACORN database it is reflected in the analysis of this study.

This study also included patients undergoing TKA for many pathologies and was not limited to osteoarthritis (OA). We did not find a significant difference in any of the three measured outcomes for patients with OA compared to all other pathologies.

An important feature of this study is that it assessed disease severity in the entire joint including the PFJ, in contrast to previous studies that only assessed the severity of changes in the TFJ with no consideration of patients who suffer from arthritis solely or predominately affecting the PFJ. Although the KL system does not include assessment of PFJ disease, we used a similar system to assign the grade of changes to PFJ and we created a disease severity grade that considered all joint compartments to account for the deficiencies in the KL system. A strength of this study is that we performed the same analysis using the original KL grading system and found similar results for all clinical outcomes. However, the novel nature of the Disease Severity grading system means that its validity and reliability are untested. The K-coefficient for interobserver reliability was acceptable (0.73) after assessing a small sample of patients. In similar studies the Kappa value for the assessment of the Kellgren–Lawrence grade is usually > 0.8. The interobserver agreement was not measured again at the end of the study. We expect that the Kappa value would be substantially higher after assessment of the significant number of patient radiographs in this study. Thus, the generalizability of the findings from this study may be limited; and future studies are needed to validate the results.

This study has several limitations; chief amongst them is the limited, short-term follow-up which is mainly related to the limited follow-up conducted by the ACORN registry. It is well recognised that TKA patients continue to improve beyond 6 months post-surgery. It is likely that the group of patients who report dissatisfaction and poor knee function at 6 months would be smaller by 12 and 24 months after TKA. This is perhaps reflected in the findings that the difference in OKS between the groups was small and not clinically significant, i.e. dissatisfaction and lack of improvement was reported despite good objective function.

Another limitation is that our definitions of the satisfied and improved groups were too wide and that a tighter definition considering only patients who were ‘Very good’; or ‘Excellent’ as satisfied and those who reported ‘Much better’ improvement in the condition of their knee as the improved group may show better discrimination between those who benefit from TKA and those who do not especially, considering the health and financial burden of the procedure. Predictive modelling of this study showed limited diagnostic ability [32] of the regression model, which meant that while statistically significant relationships were found, the use of these models as pre-operative predictive tools is associated with poor capacity to discriminate between patients who may be at higher risk of reporting dissatisfaction or failure to improve after TKA.

This study included 72% of eligible patients. Almost 14% of eligible patients did not attend the preadmission clinic or did not have a new set of knee radiographs; and almost 14% were lost to follow-up for the collection of their 6-month outcomes. This is partly because of the opt-out nature of the ACORN registry where patients who no longer want to participate in the registry choose not to reply to follow-up attempts.

It is important to note that this study did not examine other potentially relevant factors such as pre-operative opioid use, different anaesthetic modalities, surgical approach and type of TKA prosthesis. Our group has previously reported on these factors and their contribution to patient outcomes. Different physiotherapy modalities do not appear to be a strong determinant of patient outcomes at 6 and 12 months after TKA [33–36], thus these variables were not included in this study. Obesity is often associated with lower knee outcomes compared to non-obese patients, but in the current study BMI was not found to be significant.

The findings of this study have high clinical relevance as it represents the clinical picture of management of a large cohort of knee arthroplasty patients who had TKA for various pathologies. While most patients improve after TKA, those with relatively less severe radiographic features of disease report less improvement and more dissatisfaction with the results of surgery. Therefore, clinicians should assess patients with mild grades of arthritis with regard to any potential physical or psychological causes of pain and encourage patients to persevere with joint-sparing, conservative management for as long as possible and only proceeding to TKA after educating patients about the higher risk of an unsatisfactory outcome.

## Conclusions

While performing TKA on patients with mild radiographic evidence of arthritis was associated with improvement in OKS, it was also associated with higher patient-reported dissatisfaction, less improvement and lower knee function in short-term follow up after TKA compared to patients with moderate-severe arthritis.

## Appendix

See Table 7.

**Table 7:** Unadjusted analysis of clinical outcomes of (a) patient satisfaction, (b) improvement in knee function, (c) mean 6-month post-operative OKS in relation to the severity of TFJ arthritis assessed using the Kellgren and Lawrence grading system

(a) KL grade	Dissatisfied (%) (*n* = 222)	OR	95% CI for OR		*p* value
			Lower	Upper	
1	10 (5)	5.63	2.63	12.05	< 0.001
2	14 (6)	4.00	2.10	7.60	< 0.001
3	147 (66)	2.90	2.08	4.04	< 0.001
4	51 (23)	Reference			Reference

(b) KL grade	Failure to improve (%) (*n* = 173)	OR	95% CI for OR		*p* value
			Lower	Upper	
1	10 (6)	6.89	3.18	14.91	< 0.001
2	18 (10)	6.71	3.65	12.35	< 0.001
3	103 (60)	2.37	1.64	3.43	< 0.001
4	42 (24)	Reference			Reference

(c) KL grade	Mean OKS (SD)	Mean difference	95% CI of the mean difference		*p* value
			Lower	Upper	
1	36 (9.53)	4.08	1.08	7.08	0.009
2	37 (9.57)	3.03	0.81	5.24	0.08
3	37 (8.56)	2.32	1.64	2.99	< 0.001
4	40 (7.13)	Reference			Reference
